# PathCare: Integrating Clinical Pathway Information to Enable Healthcare Prediction at the Neuron Level

**DOI:** 10.3390/bioengineering12060578

**Published:** 2025-05-28

**Authors:** Dehao Sui, Lei Gu, Chaohe Zhang, Kaiwei Yang, Xiaocui Li, Liantao Ma, Ling Wang, Wen Tang

**Affiliations:** 1 Peking University Third Hospital, Beijing 100191, China; dehaosui1@gmail.com; 2 National Engineering Research Center for Software Engineering, Peking University, Beijing 100871, China; 954552967gl@gmail.com (L.G.); choc@pku.edu.cn (C.Z.); yangkw003@gmail.com (K.Y.); lixiaocui@pku.edu.cn (X.L.); malt@pku.edu.cn (L.M.); 3 Key Laboratory of High Confidence Software Technologies, Ministry of Education, Beijing 100871, China; 4 Affiliated Xuzhou Municipal Hospital of Xuzhou Medical University, Xuzhou 221002, China

**Keywords:** electronic health record, healthcare prediction, clinical prognosis

## Abstract

Electronic Health Records (EHRs) offer valuable insights for healthcare prediction. Existing methods approach EHR analysis through direct imputation techniques in data space or representation learning in feature space. However, these approaches face the following two critical limitations: first, they struggle to model long-term clinical pathways due to their focus on isolated time points rather than continuous health trajectories; second, they lack mechanisms to effectively distinguish between clinically relevant and redundant features when observations are irregular. To address these challenges, we introduce PathCare, a neural framework that integrates clinical pathway information into prediction tasks at the neuron level. PathCare employs an auxiliary sub-network that models future visit patterns to capture temporal health progression, coupled with a neuron-level filtering gate that adaptively selects relevant features while filtering out redundant information. We evaluate PathCare on the following three real-world EHR datasets: CDSL, MIMIC-III, and MIMIC-IV, demonstrating consistent performance improvements in mortality and readmission prediction tasks. Our approach offers a practical solution for enhancing healthcare predictions in real-world clinical settings with varying data completeness.

## 1. Introduction

Electronic Health Records (EHRs) have become indispensable in modern clinical healthcare, offering a rich source of data that chronicles a patient’s medical history. Over recent years, deep learning-based prediction models have gained significant attention for their ability to leverage EHR data, which represented as temporal sequences of clinical visits, can significantly inform and enhance healthcare decision-making [1]. Such applications range from disease diagnosis and mortality prediction to patient sub-typing and personalized treatment planning [2,3,4].

Working with EHR data presents challenges due to its inherent limitations in clinical practice. Factors such as rare disease occurrences, expensive examinations, and safety considerations result in data scarcity [5]; for instance, missing or inconsistent follow-up visits disrupt the continuity of patient health trajectories, creating substantial gaps in longitudinal records [2]. A patient with diabetes may have regular check-ups for three months and then miss several appointments, resulting in incomplete monitoring of critical indicators like HbA1c levels [6]. These discontinuities create fractured representations of patient status, making it difficult to model disease progression accurately. Consequently, task-specific models trained on such incomplete datasets often suffer from overfitting to observed patterns while failing to capture true health trajectories, compromising both performance and robustness in real-world applications.

While most existing works tackling EHR data sparsity challenges can be categorized into direct data space and indirect representation space methods, they generally follow four key approaches. Direct methods include attention-based models like RETAIN [2], which identify influential visits through neural attention but focus only on available data points without addressing missing temporal contexts. Interpretability-focused approaches [3,7] enhance transparency through multichannel feature extraction and importance recalibration, yet they struggle with irregularly sampled features. Indirect methods include data scarcity solutions like multi-task learning [5], SAFARI [6], which apply correlational sparsity prior but require extensive expert annotation, and advanced representation learning techniques [8], which employ self-supervised learning but often capture patterns unrelated to the target prediction task.

Despite recent advancements, contemporary methods face the following two major limitations: (1) insufficient modeling of long-term health trajectories, particularly in contexts where follow-up visits occur at irregular intervals; and (2) inadequate ability to selectively identify features that are most relevant to specific prediction tasks. For example, consider a diabetes patient who misses several follow-up appointments; current models may not only struggle to relate the patient’s initially stable status to later complications due to gaps in the temporal sequence, but they may also fail to effectively filter out irrelevant features (such as unrelated laboratory values or demographic attributes) while emphasizing clinically significant variables that contribute to the risk of disease progression. As a result, critical trajectory patterns and key predictors may be overlooked, impairing model performance for individualized risk assessment.

Intuitively, the way clinicians approach patient care offers valuable inspiration for modeling clinical pathways. When faced with a patient who has missed follow-ups, physicians naturally try to fill in the gaps by considering the patient’s historical trends and likely progression based on similar cases they have encountered. They focus on the most predictive indicators while filtering out irrelevant information. Similarly, an effective predictive modeling approach should leverage the predictive power of historical visit patterns to anticipate likely future states, enabling a more continuous representation of the patient’s health trajectory despite missing observations. This perspective aligns with how language models predict the next word in a sentence based on context, suggesting that viewing clinical pathways as “health sentences” could yield more coherent patient representations [9].

Given these insights, we are confronted with the following pressing challenge: How can we effectively model the long-term clinical pathway to capture future-predictive health representations, despite missing or inconsistent follow-ups, while filtering out redundant information that might compromise target prediction?

To address this, we introduce PathCare with two key innovations that directly tackle the identified limitations. First, we develop a longitudinal-aware auxiliary sub-network that predicts future clinical visits and encodes health status from a temporal perspective, enabling the effective modeling of long-term clinical pathways even with missing observations. Second, we design a neuron-level filtering mechanism that adaptively selects target-predictive features while filtering out redundant information, ensuring focus on clinically meaningful signals.

Central to our approach is the view of a patient’s clinical pathway as analogous to a sentence in natural language processing, with each visit considered a word containing lab tests and events [9]. Unlike traditional methods requiring additional labeling or complex feature engineering [5], our auxiliary sub-network evaluates the reliability of each feature by analyzing historical visit patterns and temporal dependencies among clinical variables. By examining how well certain features predict future health states, our model learns which aspects of the patient record are most informative for clinical outcomes, even when observations are irregular or missing.

Our neuron-level filtering mechanism, based on layer decorrelation, encourages diversity among hidden units while adaptively selecting target-predictive features. High-ranking neurons that capture essential health progression patterns are preserved, while low-ranking neurons that model noise or redundant information are filtered out. This approach mimics how physicians focus on key indicators while disregarding less relevant measurements [6,10].

We argue that jointly learning future-predictive health representations offers valuable insights for prognosis, beyond independently predicting target labels. For example, when predicting diabetes risk, incorporating blood glucose level predictions as an auxiliary task enhances the primary task by capturing the underlying progression pattern. Unlike previous multi-task approaches with parameter hard-sharing [5], PathCare provides these as separate hidden features, avoiding interference with the primary task while benefiting from the shared information.

Our primary contributions are outlined as follows:

- Methodologically, we propose PathCare, a framework for learning future-predictive health representations, designed to model long-term clinical pathways despite missing follow-ups. We design a longitudinal-aware auxiliary sub-network that predicts future clinical visits and encodes health status from a temporal perspective. We also introduce a neuron-level filtering mechanism that adaptively selects target-predictive features, encouraging diversity among hidden units while preserving critical information. PathCare provides refined longitudinal modeling and feature selection, with disrupted health trajectories elaborately modeled, resulting in more robust patient representations across irregular visit patterns.
- Experimentally, evaluations on CDSL, MIMIC-III, and MIMIC-IV datasets demonstrate PathCare’s superior performance in mortality and readmission prediction tasks, achieving relative AUPRC improvements of up to 2.43% and consistently higher min(+P, Se) values across all datasets. Our model maintains robust performance even under extreme data sparsity conditions and shows particular effectiveness for patients with regular missing visits. Ablation studies confirm that both the pathway prediction component and adaptive filtering mechanism contribute significantly to performance gains, validating our approach to clinical trajectory modeling in real-world settings with varying data completeness.

## 2. Related Work

In the field of EHR data analysis, data sparsity caused by irregular sampling poses significant challenges for building efficient prediction models [11,12]. Previous approaches primarily fall into the following two categories: methods that directly address sparsity in the data space and methods that indirectly resolve sparsity in the representation space.

### 2.1. Direct Data Space Methods

Direct data space methods aim to operate directly on raw EHR data through feature extraction or data imputation to address missing value problems. Traditional approaches rely on imputation techniques [13] or statistical methods [14], which assume patient visits are independent and features are missing randomly, ignoring the fact that missingness in EHR data often contains important clinical information. More sophisticated architectures like RETAIN [2] use two-level attention to identify important visits and variables but neglect the fine-grained importance of feature changes. While RETAIN [2] identifies influential visits and variables, its primary focus on available data points makes it challenging to infer progression along an implicit clinical pathway, especially when significant temporal gaps arise from irregular follow-ups. The attention mechanism may not fully capture the underlying clinical logic connecting temporally distant but pathologically related events if the intervening pathway segments are unobserved or poorly represented due to sampling irregularities. Recent models [3,15,16] enhance representation through time-aware distributions, multi-scale feature extraction, and adaptive feature importance recalibration, but they still face fundamental challenges with highly sparse data—extracting sufficient information from limited observations to construct complete representations.

The core limitation of direct methods is their reliance solely on limited data from individual patients, ignoring valuable information in cross-patient patterns, making it difficult to construct complete representations in highly sparse environments. Furthermore, even state-of-the-art feature extraction techniques struggle with high proportions of missing values, especially when the missing patterns themselves contain clinical information [17].

### 2.2. Indirect Representation Space Methods

Indirect representation space methods address sparsity problems in the representation space by utilizing auxiliary information or similar patient data, rather than directly filling in missing values in the original data [5].

One direction leverages patient similarities. GRASP [4] finds similar patients to enhance representation learning, while other approaches [10] compensate for missing modality information using auxiliary data from similar patients. However, these methods underestimate the impact of missing features when measuring patient similarity, leading to inaccurate similarity assessments. More importantly, they focus on how to extract information from similar patients, rather than how to effectively integrate this information.

Some methods enhance representations through multi-task learning [5,17]. Current approaches often employ parameter hard-sharing strategies for multi-task models, failing to explicitly consider what to share and how much to share it, which can lead to negative transfer that interferes with target prediction optimization.

Some recent works [6,8] learn self-supervised representations for irregular time series through time-sensitive contrastive learning and data reconstruction, but they fail to fully utilize the implicit clinical pathway information in EHR data, which is crucial for capturing dynamic changes in patients’ health status. For instance, advanced sequential models like Mamba [9], while effective at capturing long-range dependencies, are not inherently designed to decode the implicit clinical pathway logic from sequences of medical codes and measurements, particularly when these sequences are characterized by irregular and unpredictable time intervals. Their state-space mechanisms might summarize long histories but may not explicitly model the ’expected next phase’ of a clinical pathway or differentiate signals critical to pathway progression from general temporal patterns, especially when faced with high data irregularity.

Overall, a significant gap persists, outlined as follows: existing methods, whether direct or indirect, often lack dedicated mechanisms to holistically interpret implicit clinical pathways from irregularly sampled EHR time series. They may either focus too narrowly on observed points, fail to adequately model the inherent pathway logic, or lack adaptive filtering robust enough for severe irregularity and missingness. This underscores the need for novel frameworks that can explicitly learn from pathway progression cues and selectively utilize features, even amidst such data imperfections.

In this paper, we propose PathCare, a general healthcare prediction model designed to address these specific challenges by integrating clinical visit pathway information. Its core innovation lies in designing a visit pathway representation learning mechanism that captures temporal dynamics of patient visit sequences and learns from similar patients through an adaptive information sharing strategy. Unlike direct methods, PathCare does not rely on the complete imputation of original data; compared to indirect methods, PathCare explicitly models what to share and how much to share from auxiliary representations, effectively mitigating negative transfer risk. In this way, PathCare provides more accurate predictions, even under highly sparse EHR data conditions.

## 3. Preliminary

In this section, we begin with a motivating example, then describe the structure of EHR data, and formulate the problem of clinical healthcare prediction.

### 3.1. A Motivating Example

We consider the health status prediction of patients with chronic or critical conditions as the motivating example. Many individuals worldwide suffer from such conditions, facing significant health risks that require ongoing treatment and periodic hospital visits for various tests (e.g., blood tests and vital sign monitoring). The accurate prediction of patient health risks based on medical records collected during these visits is crucial in supporting recovery and preventing adverse outcomes.

### 3.2. Problem Formulation

**Figure 1:**
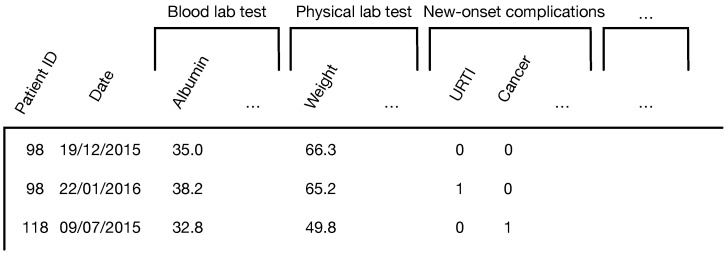
Time-series data of EHR. Patients may visit the hospital multiple times. The health status is depicted by various clinical features, including numerical variables (e.g., lab tests) and categorical variables (e.g., diagnosis codes).

EHR data are routinely collected from patient observations in hospitals through clinical visits, encompassing discrete time-series data (e.g., medication and diagnosis) and continuous multivariate data (e.g., vital signs and laboratory measurements). We assume a patient visits the clinic *t* times, generating time-ordered EHR records denoted as $r_{t}\in R^{N_{r}}$(t=1,2,⋯,T). Each EHR record contains $N_{r}$ features, such as lab test results or clinical observations, as illustrated in Figure 1. The prediction problem in this paper is formulated as follows: given *t* historical EHR records of a patient, i.e., $\left(r_{1},\cdots ,r_{t}\right)$, how can we predict the patient’s healthcare status *y*, which represents the probability of encountering a specific risk (e.g., in-hospital mortality or readmission). The next section will detail the proposed model. Table 1 lists the notations used in our model.

## 4. Method

**Figure 2:**
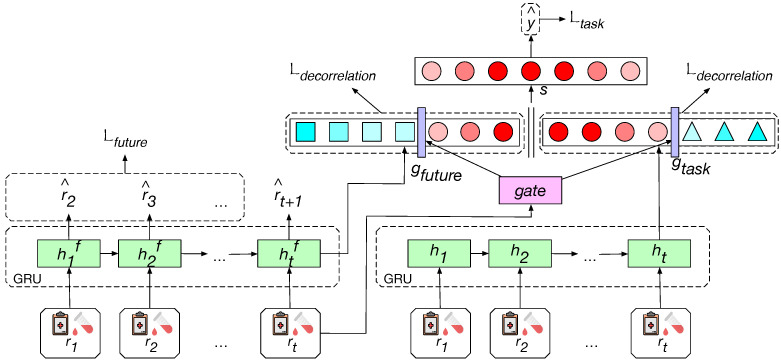
Framework of PathCare. The embedding extracted to predict the future visit, $h_{t}^{f}$, is provided as additional advanced clinical features and combined with task-specific embedding $h_{t}$. A neuron-level filtering gate is designed to adaptively order the neurons by modeling the demand degree of clinical pathway prospects for patients in diverse conditions. High-ranking neurons (denoted as red circles) will store target-predictive information, which is expected to be kept, while low-ranking neurons will store target-irrelevant information that should be filtered out (denoted as blue squares and triangles).

For the healthcare prediction task, the model is trained to utilize the recorded clinical visits ($r_{t}\in R^{N_{r}}$) to learn the representation of health status and predict the probability of suffering from the specific target outcome in the future $\hat{y}$ (e.g., the risk of in-hospital mortality or readmission). In this paper, we propose a general model, PathCare (illustrated in Figure 2), which adds useful auxiliary embedding to healthcare prediction tasks by predicting future visits.

- We explicitly model the clinical status pathway by training a Gated Recurrent Unit (GRU)-based auxiliary sub-network (i.e., the left GRU) to predict the lab tests and clinical events recorded in a future visit (${\hat{r}}_{t+1}$). The hidden representation of the sequence ($h_{t}^{f}$) is encoded to be a good predictor of future status, and it is provided as extra clinical features for the supervised clinical prediction task. This helps the model to depict the health status from a long-term perspective.
- A task-specific GRU is applied to extract the other part of the health status representation. The model merges the task-specific representation and the auxiliary representation to perform the target prediction. We encourage the diversity among hidden units based on layer decorrelation to help the useful units stand out (i.e., denoted as red circles). A neuron-level gate is designed to filter out the units that are useless to the target prediction (i.e., denoted as blue squares on the left side, and blue triangles on the right side) and reduce the redundancy of the model.

### 4.1. Auxiliary Task for Clinical Pathway Modeling

For the sake of causality, the model is not allowed to consult the lab tests and clinical events recorded in future visits and take them as input (i.e., clinical features), while, in reality, they are closely related to the target outcome in the future. It is beneficial to jointly model the clinical pathway in the future instead of independently predicting the target label. However, it may introduce redundancy and disturb the optimization of the original network if the model is made to predict the future visit and target outcome by directly performing multi-task learning in the same model. Inspired by [18] in natural language processing, which adds the context meaning of words to the sequence tagging model by pre-training an extra language model, we construct a separate auxiliary network to predict lab tests and clinical events in the future. The sub-network takes the patient’s EHR records $r_{t}\in R^{N_{r}}$(t=1,2, ⋯ ,T) as the input and embeds the time sequence with a GRU [19],

(1) $$h_{1}^{f},\ldots ,h_{T}^{f}={\mathrm{GRU}}_{\mathrm{future}}\left(r_{1},\ldots ,r_{T}\right),$$

where $h_{t}^{f}\in R^{N_{h}}$(t=1,2,⋯,T) represents the learned representations for each clinical visit, and $N_{h}$ is the hidden dimension. These representations are called future-specific representations, and they contain information on the future status of the patients since they are used to predict future events in the model. Then, those representations are fed into a feedforward layer to predict the lab test values of the next clinical visit,

(2) $${\hat{r}}_{t+1}=\mathrm{FNN}\left(h_{t}^{f}\right).$$

We do not need extra labels but use the time sequence itself to supervise the training. We calculate the mean-squared loss for every predicted visit except the last predicted one, whose ground-truth value is not recorded in the data.

(3) $$L_{future}=\frac{1}{T}\sum_{t=1}^{T-1}\mathrm{MSE}\left({\hat{r}}_{t+1},r_{t+1}\right).$$

The extracted hidden embeddings for the sequence will be used as additional clinical features in the supervised target prediction model. In particular, we concatenate the embedding ($h_{t}^{f}$) with the output from the GRU layers in the target prediction network ($h_{t}$). However, $h_{t}^{f}$ is trained to predict the future clinical visit and thus contains a lot of target-irrelevant information, which may introduce undesired bias that can degrade the performance of target prediction. To exploit the beneficial information for target prediction, we force neurons to represent different information and filter out the target-irrelevant ones based on the neuron-level filtering gate in the next subsection.

### 4.2. Neuron-Level Filtering Gate

The task-specific module takes the patient’s raw EHR records $r_{t}\in R^{N_{r}}$(t=1,2,⋯,T) as the input and embeds the time sequence with a task-specific GRU,

(4) $$h_{1},\ldots ,h_{T}={\mathrm{GRU}}_{\mathrm{task}}\left(r_{1},\ldots ,r_{T}\right),$$

where $h_{t}\in R^{N_{h}}$(t=1,2, ⋯ ,T) represents the task-specific representations for each clinical visit, and $N_{h}$ is the hidden dimension. These representations are called task-specific representations, and they contain task-specific information of the patients. The future-specific representations and task-specific representations are then projected into a new latent space,

(5) $$\begin{array}{c} g_{future}=W_{f}h_{t}^{f}\\ g_{task}=W_{t}h_{t} \end{array}$$

where $W_{f}\in R^{N_{g}\times N_{h}}$ and $W_{t}\in R^{N_{g}\times N_{h}}$ are the projection matrices for the two different representations. We construct a combined system, using $g_{future}$ as additional advanced clinical features. A simple method is to concatenate $g_{future}$ with $g_{task}$. To effectively exploit information that is beneficial for the target prediction in auxiliary embeddings and reduce the redundancy, we intend to use a neuron-level filtering gate, which adaptively models the demand degree of clinical pathway information for patients in different conditions and filters out useless neurons.

Firstly, we promote diversities among hidden neurons (i.e., differentiation of information stored inside each neuron) to prepare for neuron-level filtering. This makes it easy to identify the target-irrelevant part. Based on [20], which uses a regularizer to reduce overfitting and increase generalization, we explicitly encourage non-redundant representations by reducing the correlation between activations in $g_{future}$ and $g_{task}$, respectively. Here, for simplicity, we use g to denote them as a general case. The covariances between all pairs of activations *i* and *j* of g form a matrix C,

(6) $$C_{i,j}=\frac{1}{B}\sum_{b=1}^{B}\left(g_{i}^{b}-\mu_{i}\right)\left(g_{j}^{b}-\mu_{j}\right)$$

where *B* is the batch size and $g_{i}^{b}$ is the *i*-th activation of g at the *b*-th case in the batch. $\mu_{i}=\frac{1}{B}\sum_{b=1}^{B}g_{i}^{b}$ is the sample mean of activation *i* over the batch. The diagonal of C is then subtracted from the matrix norm to build the decorrelation loss term as follows:

(7) $$L_{decorrelation}=\frac{1}{2}{(∥C∥}_{F}^{2}-{∥\mathrm{diag}\left(C\right)∥}_{2}^{2})$$

where ${∥\cdot ∥}_{F}$ is the Frobenius norm, and the diag(·) operator extracts the main diagonal of a matrix into a vector. The decorrelation loss will co-operate with the target prediction loss to decompose the target-relevant part from the irrelevant part, which helps to ease the difficulty in mining useful information.

Secondly, we propose a filtering gate to model the demand degree of future visit prediction for patients in diverse conditions. If the gate believes that jointly evaluating the future visit is beneficial to the target prediction for a patient, more hidden units in auxiliary embeddings will be included in the final health status representation. Otherwise, the gate will tend to select the hidden units in task-specific embeddings. Only the neurons selected by the gate are used to perform the target prediction. Concretely, the filtering gate is learned based on the latest record of the patient,

(8) $$\begin{array}{c} \mathrm{gate}=W_{gate}r_{t} \end{array}$$

where $W_{gate}\in R^{N_{g}\times N_{r}}$ is the projection matrix for the record in the last time step. Inspired by [21], which proposes a novel inductive bias for recurrent neural networks to separately allocate hidden state neurons with long and short-term information, we use the similar cumax function to operate the learned gate and obtain the valve for $g_{future}$ and $g_{task}$.

(9) $$\begin{array}{c} \mathrm{cumax}(...)=\mathrm{cumsum}(\mathrm{softmax}(...))\\ {\mathrm{gate}}_{future}=\mathrm{cumax}\left(\mathrm{gate}\right)\\ {\mathrm{gate}}_{task}=1-\mathrm{cumax}\left(\mathrm{gate}\right) \end{array}$$

where cumsum denotes the cumulative sum. The output of vector cumax can be seen as the expectation of a binary gate (0,…,0,1,…,1) [21]. This binary gate splits the cell state into two segments as fiollows: the 0-segment and the 1-segment. Thus, ${\mathrm{gate}}_{future}$ and ${\mathrm{gate}}_{task}$ act as complementary gate vectors, where ${\mathrm{gate}}_{task}=1-{\mathrm{gate}}_{future}$ element-wise, ensuring they operate on distinct neural pathways. We obtain a new state representation by using the learned gate to adaptively extract information from the future/task-specific embeddings,

(10) $$\begin{array}{c} s={\mathrm{gate}}_{future}⊙g_{future}+{\mathrm{gate}}_{task}⊙g_{task} \end{array}$$

where ⊙ represents element-wise multiplication. The patient’s state representation s is fed into a feedforward layer to predict the final task,

(11) $$\hat{y}=\mathrm{FNN}\left(s\right).$$

The corresponding target prediction loss and the final optimization loss are, respectively, defined as

(12) $$L_{task}=-\frac{1}{N}\sum_{i=1}^{N}\left(y_{i}\log\left({\hat{y}}_{i}\right)+\left(1-y_{i}\right)\log\left(1-{\hat{y}}_{i}\right)\right)$$

(13) $$L_{total}=L_{task}+\alpha L_{decorrelation}$$

where α is a hyper-parameter. The synergistic operation of these components—specifically, the initial learning of future-specific and task-specific representations in separate GRUs; the promotion of internal diversity within these projected representations ($g_{future}$, $g_{task}$) via the $L_{decorrelation}$ loss; and critically, the adaptive neuron-level filtering gate—plays a crucial role in mitigating the risk of negative transfer. By dynamically selecting and weighting neuronal information from both the future-predictive and task-specific embeddings based on the current input instance ($r_{t}$), PathCare can effectively filter out potentially irrelevant or conflicting signals that might originate from the auxiliary future-prediction task. This adaptive control ensures that predominantly beneficial information contributes to the final health status representation s and the primary prediction $\hat{y}$.

## 5. Experimental Setups

### 5.1. Datasets

We utilize the CDSL (COVID Data Save Lives), MIMIC-III, and MIMIC-IV datasets for benchmarking, with detailed statistics presented in Table 2 and Table 3.

CDSL dataset [22]. This dataset contains anonymized records of 4255 COVID-19 patients from Spain’s HM Hospitales [22]. The CDSL dataset is only used for mortality prediction as readmission information is unavailable.

MIMIC-III dataset (version 1.4) [11]. MIMIC-III contains de-identified health data from over 40,000 ICU patients (2001–2012), including demographics, vital signs, laboratory tests, and medications.

MIMIC-IV dataset (version 2.2) [12]. This updated version collected from 2008 to 2019 includes 24,610 samples after preprocessing, with approximately 29.5% positive samples for both mortality and readmission.

For all datasets, we apply the same preprocessing, outlined as follows: (1) forward filling missing values with patients’ most recent records; (2) standardizing features to zero mean and unit variance; (3) imputing missing features with dataset-level averages when all records are missing. We strictly maintain causality throughout preprocessing to ensure test data integrity.

For the MIMIC-III dataset, the split of 80% training, 15% validation, and 5% testing was chosen to strictly align with the cohort selection and data splitting approach used in the benchmark study by Harutyunyan et al. [5]. This adherence ensures that our performance evaluation on MIMIC-III is directly comparable to established prior work. The specific data splits for CDSL (70% training, 10% validation, and 20% testing) and MIMIC-IV (70% training, 10% validation, and 20% testing) represent a standard and robust partitioning for machine learning model development and evaluation. For mortality prediction, we use the first 48 h of ICU data; for readmission prediction, we use all data before discharge.

### 5.2. Evaluation Metrics

We assess model performance using AUPRC, AUROC, and min(+P, Se). For our imbalanced datasets, AUPRC provides more informative assessment than AUROC, as it emphasizes positive class performance [23]. The min(+P, Se) metric, the minimum of precision (+P) and sensitivity (Se, or recall), ensures there is a balance between accurate positive predictions and capturing true positives. This is vital in healthcare, where both minimizing false alarms and detecting true cases matter.

### 5.3. Prediction Tasks

We conduct experiments on the following two clinically relevant prediction tasks.

Mortality prediction. This task predicts whether a patient will die during hospitalization. For the MIMIC datasets, we use the first 48 h of ICU data to predict in-hospital mortality (12% positive cases). For the CDSL dataset, we predict COVID-19 mortality (12.69% positive cases), which is particularly challenging due to the disease’s novel nature and varied impact across populations.

Readmission prediction. The 30-day readmission prediction task aims to predict hospital readmission within 30 days after discharge. Available only for MIMIC datasets, this task uses all pre-discharge data, with approximately 16% positive samples in MIMIC-III and 15.5% in MIMIC-IV.

Both tasks have significant clinical value for identifying high-risk patients, optimizing resource allocation, and enabling early interventions.

### 5.4. Baseline Models

We compare PathCare with several state-of-the-art approaches as follows:

#### 5.4.1. EHR-Specific Models

- RETAIN [2] utilizes a two-level neural attention mechanism to detect influential visits and significant clinical variables within those visits. It processes EHR data in reverse time order, mimicking physician practice by giving higher attention to recent clinical visits.
- SAFARI [6] learns compact patient health representations by imposing a correlational sparsity prior to the correlations of medical feature pairs. It solves a bi-level optimization problem involving high-level inter-group correlations and lower-level intra-group correlations, using Laplacian kernel and graph neural networks.
- AdaCare [16] captures the long- and short-term variations of biomarkers as clinical features to represent health status across multiple time scales. It models correlations between clinical features to enhance those that strongly indicate health status, maintaining high prediction accuracy while providing interpretability.
- GRASP [4] enhances patient representation learning by leveraging knowledge from similar patients. It defines similarities between patients for different clinical tasks, finds similar patients with useful information, and learns cohort representation to extract valuable knowledge.

#### 5.4.2. General Deep Learning Models

- RNN is a standard recurrent neural network model applied to sequential medical data, serving as a fundamental baseline.
- GRUα is a basic GRU model with an addition-based attention mechanism, serving as a strong baseline for healthcare prediction tasks.
- Mamba [9] is a linear-time sequence modeling architecture based on selective state spaces. It allows the model to selectively propagate or forget information along the sequence length dimension, depending on the current token, making it suitable for processing long sequences of medical data.

#### 5.4.3. Ablation Models

To evaluate our model components, we include the following two PathCare variants:

- ${\mathrm{PathCare}}_{Context-}$ removes the future clinical pathway context module, focusing solely on current information for prediction.
- ${\mathrm{PathCare}}_{Gate-}$ directly concatenates auxiliary and task-specific embeddings without the neuron-level filtering gate, maintaining potential redundancy between embeddings.

### 5.5. Implementation Details

Hardware and software. All models are trained on a single NVIDIA RTX 3090 GPU with CUDA 11.8 and 64 GB system memory. We implement our method using Python 3.11.4, PyTorch 2.0.1 [24], PyTorch Lightning 2.0.5 [25], and pyehr [26,27].

Training and hyperparameters. We use AdamW optimizer [28] with a batch size of 256 patients for all models. Training runs for a maximum of 100 epochs with early stopping after 10 epochs without AUPRC improvement on the validation set. We employ a learning rate of 0.001 with linear warmup (first 5 epochs) followed by cosine decay.

To ensure reproducibility, we fix the random seed to 42 for all experiments and employ bootstrapping on the test sets for robust performance evaluation.

## 6. Experimental Results and Analysis

We evaluate PathCare on in-hospital mortality prediction tasks across CDSL, MIMIC-III, and MIMIC-IV datasets, as well as 30-day readmission prediction tasks on MIMIC-III and MIMIC-IV datasets.

### 6.1. Experimental Results

Table 4 and Table 5 present performance comparisons between PathCare and the existing methods across multiple datasets and prediction tasks.

PathCare consistently outperforms the baseline methods across all datasets and tasks. For mortality prediction (Table 4), PathCare surpasses the strongest baselines by 0.5–1.3% in AUPRC and 0.5–1.6% in AUROC across datasets. More substantial improvements are observed in the min(+P, Se) metric, with gains of 4–22% over the best-performing baselines, demonstrating PathCare’s superior ability to balance precision and sensitivity.

For readmission prediction (Table 5), performance advantages become more pronounced. PathCare improves AUPRC by 0.5–0.8% and AUROC by 0.2–0.3% over the best baselines. The most significant gains occur in min(+P, Se), where PathCare outperforms existing methods, highlighting its particular strength in maintaining clinically valuable prediction balance.

Comparative analysis reveals that PathCare addresses some of the fundamental limitations in the existing approaches. Direct data space methods (RETAIN, AdaCare) rely solely on patient-specific observations without leveraging cross-patient information patterns. Indirect representation space methods (GRASP) lack explicit mechanisms for determining optimal information sharing strategies. Even advanced sequential modeling approaches like Mamba underperform compared to PathCare, underscoring the importance of both temporal modeling and contextual information integration for effective healthcare prediction.

### 6.2. Ablation Study

To understand component contributions, we compare PathCare with the following two variants: ${\mathrm{PathCare}}_{Context-}$ (without future clinical pathway context) and ${\mathrm{PathCare}}_{Gate-}$ (without a neuron-level filtering gate). The results in Table 4 and Table 5 show both variants achieve competitive performance against the baselines but are outperformed by the complete PathCare.

${\mathrm{PathCare}}_{Context-}$ performs worse than the full model, with notable AUPRC decreases in mortality prediction (1.55% for CDSL and 6.01% for MIMIC-III) and readmission prediction (3.88% for MIMIC-III and 4.98% for MIMIC-IV). This confirms that modeling future clinical pathways provides valuable information for health status assessment and prediction.

Similarly, ${\mathrm{PathCare}}_{Gate-}$ shows performance drops across datasets, with AUPRC decreases of 2.00%, 3.37%, and 2.33% for mortality prediction on CDSL, MIMIC-III, and MIMIC-IV, respectively, and 3.95% for MIMIC-III readmission. These results demonstrate the efficacy of the neuron-level filtering gate in reducing redundancy and selecting relevant information from auxiliary embeddings.

These findings confirm that both future clinical pathway modeling and neuron-level filtering mechanisms are essential components of PathCare, contributing to its superior performance in healthcare prediction tasks.

### 6.3. Observations and Analysis

**Figure 3:**
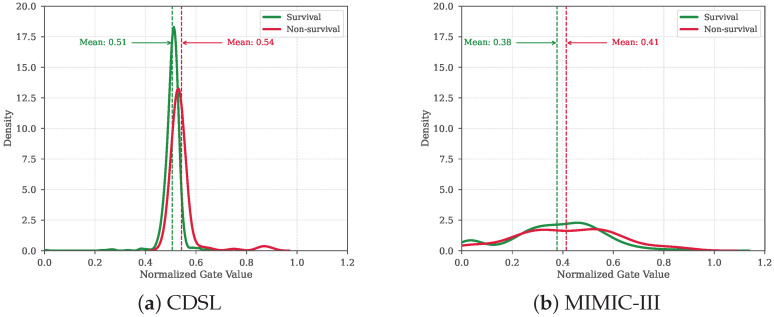
Distribution of gate values for survival vs. non-survival groups across datasets. Higher values indicate greater reliance on future-predictive features.

To further understand how PathCare adaptively selects information from future-predictive embeddings versus task-specific embeddings, we examine the learned gate values across different patient groups. Figure 3 illustrates the distribution of gate values for survival and non-survival groups across the CDSL and MIMIC-III datasets.

Interestingly, patients with adverse outcomes (non-survival) consistently exhibit higher average gate values compared to those with favorable outcomes (survival) across both datasets (CDSL: 0.54 vs. 0.51; MIMIC-III: 0.41 vs. 0.38). This indicates that PathCare adaptively allocates more representation capacity to future-predictive features for high-risk patients, while relying more on task-specific features for low-risk patients.

For patients at higher risk, the model appears to emphasize capturing recent deterioration patterns and near-term trajectory shifts that signal potential complications. Conversely, for patients with better prognoses, the model incorporates more auxiliary embeddings to enhance representation robustness, potentially accounting for the greater stability and predictability in their clinical trajectories. This adaptive mechanism aligns with clinical practice, where physicians pay closer attention to immediate risk signals in critically ill patients while considering broader health indicators for stable patients.

This adaptive reliance on future-predictive features, controlled by the gate mechanism, as evidenced by the distinct distributions in Figure 3, highlights how PathCare can strategically leverage auxiliary information when it is deemed beneficial (e.g., for high-risk patients), while simultaneously down-weighting its influence when it might be less relevant or potentially introduce noise for other patient groups. Such dynamic information flow control is a key aspect of the model’s strategy to mitigate negative transfer from the auxiliary task, ensuring robust performance across diverse patient conditions.

## 7. Conclusions

In this paper, PathCare is proposed to perform healthcare prediction by integrating clinical pathway information at the neuron-level. Specifically, an auxiliary sub-network is built to predict future clinical visits and provide additional context-sensitive information for the primary prediction task. We encourage diversities among hidden units in the health status embedding based on layer decorrelation to help the target-relevant units stand out. We design a neuron-level filtering gate to order the neurons and adaptively select the target-predictive units to predict the primary target for patients in diverse conditions. Experimental results on the MIMIC-III, MIMIC-IV, and CDSL datasets demonstrate that PathCare consistently outperforms state-of-the-art baseline approaches across various healthcare prediction tasks. Our work provides a new perspective on integrating clinical pathway information for improved healthcare prediction, which could potentially assist healthcare providers in making more informed decisions for patient care. Online Resources in Appendix A.

## Figures and Tables

**Table 1:** Notations for PathCare.

Notation	Definition
$y_{t}$	Ground truth of prediction target at *t*-th visit
${\hat{y}}_{t}$	Prediction result at *t*-th visit
$r_{t}\in R^{N_{r}}$	Multivariate visit record at *t*-th visit
${\hat{r}}_{t+1}$	Prediction result of the status of the next visit
$h_{t}^{f}\in R^{N_{h}}$	Health status embedding learned to predict the future visit
$h_{t}\in R^{N_{h}}$	Health status embedding learned to predict the primary target
$g_{future}\in R^{N_{g}}$	Projection of future-specific embedding
$g_{task}\in R^{N_{g}}$	Projection of task-specific embedding
$\mathrm{gate}\in R^{N_{g}}$	Neuron-level gate for modeling the demand degree of a future visit
${\mathrm{gate}}_{future}\in R^{N_{g}}$	Mask for selecting units from $g_{future}$
${\mathrm{gate}}_{task}\in R^{N_{g}}$	Mask for selecting units from $g_{task}$
$s\in R^{N_{g}}$	Combined health status representation for the target prediction
*B*	Batch size
$C_{i,j}$	Covariances between all pairs of activations *i* and *j* in the layer
$g_{i}^{b}$	The *i*-th activation of g at the *b*-th case in the batch
$\mu_{i}$	The sample mean of activation *i* over the batch
$W_{f}$	Projection matrix for future representations
$W_{t}$	Projection matrix for task-specific representations
$W_{gate}$	Projection matrix for the filtering gate
α	Hyperparameter for the decorrelation loss term
$L_{future}$	Loss for predicting the next visit
$L_{decorrelation}$	Decorrelation loss term
$L_{task}$	Loss for the primary prediction task

**Table 2:** Statistics of the experimented datasets after preprocessing. The # Samples column shows the number of samples and their percentage of the entire dataset, indicating data splits (train, val, and test). The # ${\mathrm{Label}}_{Out.}=1$ and # ${\mathrm{Label}}_{Re.}=1$ columns provide the count and percentage of patients with adverse outcomes within each data split. “Out.” denotes “mortality outcome”, and “Re.” denotes “Readmission”.

Dataset	Split	# Samples	# ${\mathrm{Label}}_{\mathrm{Out}.}=1$	# LabelRe.=1
CDSL	Train	2978 (69.99%)	378 (12.69%)	-
	Val	426 (10.01%)	54 (12.68%)	-
	Test	851 (20.00%)	108 (12.69%)	-
MIMIC-III	Train	16,094 (80.00%)	4996 (31.04%)	4996 (31.04%)
	Val	3018 (15.00%)	934 (30.95%)	934 (30.95%)
	Test	1006 (5.00%)	312 (31.01%)	312 (31.01%)
MIMIC-IV	Train	17,227 (70.00%)	5095 (29.58%)	5095 (29.58%)
	Val	2461 (10.00%)	724 (29.42%)	724 (29.42%)
	Test	4922 (20.00%)	1450 (29.46%)	1450 (29.46%)

**Table 3:** Detailed statistics of the CDSL dataset.

Statistic	Total	Survived	Deceased
Number of patients	4255	3715 (87.31%)	540 (12.69%)
Number of records	123,044	108,142 (87.89%)	14,902 (12.11%)
Records per patient	24.0 [15, 39]	25.0 [15, 39]	22.5 [11, 37]
Age	67.2 [56.0, 80.0]	65.1 [54.0, 77.0]	81.6 [75.0, 89.0]
Age > Mean (67 years)	2228 (52.36%)	1748 (47.05%)	480 (88.89%)
Age ≤ Mean (67 years)	2027 (47.64%)	1967 (52.95%)	60 (11.11%)
Male	2515 (59.11%)	2173 (58.49%)	342 (63.33%)
Female	1740 (40.89%)	1542 (41.51%)	198 (36.67%)
Number of features	99		
Length of stay (days)	6.4 [4.0, 11.0]	6.1 [4.0, 11.0]	6.0 [3.0, 10.0]

**Table 4:** Performance comparison of different methods on mortality prediction tasks across the CDSL, MIMIC-III, and MIMIC-IV datasets.

Methods	CDSL Mortality			MIMIC-III Mortality			MIMIC-IV Mortality		
	AUPRC (↑)	AUROC (↑)	min(+P, Se) (↑)	AUPRC (↑)	AUROC (↑)	min(+P, Se) (↑)	AUPRC (↑)	AUROC (↑)	min(+P, Se) (↑)
RETAIN	77.23 ± 4.13	93.67 ± 1.56	68.96 ± 4.14	45.60 ± 4.48	83.89 ± 2.12	28.74 ± 4.27	51.53 ± 1.40	**86.61 ± 0.55**	33.09 ± 1.77
RNN	83.03 ± 2.97	95.55 ± 0.82	69.02 ± 5.06	47.63 ± 5.86	84.03 ± 1.93	28.73 ± 4.97	51.92 ± 1.67	85.09 ± 0.71	30.92 ± 1.42
SAFARI	76.70 ± 4.02	94.42 ± 1.27	63.96 ± 3.99	48.32 ± 3.08	84.57 ± 0.94	25.31 ± 1.94	49.25 ± 1.88	85.21 ± 0.83	32.22 ± 1.73
AdaCare	82.10 ± 4.02	94.78 ± 1.19	72.57 ± 3.60	51.19 ± 2.90	83.72 ± 0.86	25.76 ± 2.44	51.18 ± 1.53	83.79 ± 0.68	34.33 ± 1.60
GRASP	83.60 ± 3.10	95.05 ± 0.96	71.12 ± 4.50	45.64 ± 6.29	83.24 ± 1.90	30.22 ± 5.81	52.63 ± 1.38	86.23 ± 0.61	30.69 ± 1.33
${\mathrm{GRU}}_{\alpha }$	80.57 ± 3.83	95.31 ± 1.13	66.38 ± 3.51	52.24 ± 2.62	85.49 ± 0.72	24.69 ± 2.48	54.61 ± 1.37	86.16 ± 0.73	42.92 ± 1.68
Mamba	79.21 ± 3.73	92.28 ± 2.06	66.69 ± 4.73	51.33 ± 3.12	85.33 ± 0.89	26.05 ± 2.08	51.66 ± 1.32	84.29 ± 0.72	32.35 ± 1.95
${\mathrm{PathCare}}_{\mathrm{Context}-}$	82.56 ± 3.03	95.74 ± 0.93	72.48 ± 3.48	47.50 ± 4.83	83.99 ± 1.62	46.40 ± 0.42	51.67 ± 4.54	85.19 ± 1.70	52.47 ± 3.67
${\mathrm{PathCare}}_{\mathrm{Gate}-}$	82.11 ± 3.73	95.24 ± 1.13	74.72 ± 3.69	50.14 ± 4.68	85.36 ± 1.65	50.80 ± 0.38	51.86 ± 4.09	84.13 ± 1.73	51.76 ± 3.39
PathCare	**84.11 ± 3.18**	**96.08 ± 0.97**	**76.55 ± 3.68**	**53.51 ± 4.40**	**85.63 ± 1.62**	**52.62 ± 0.16**	**54.19 ± 1.86**	85.91 ± 0.65	**52.62 ± 1.56**

**Note:** Bold values indicate the best performance in each column.

**Table 5:** Performance comparison of different methods on 30-day readmission prediction tasks across the MIMIC-III and MIMIC-IV datasets.

Methods	MIMIC-III Readmission			MIMIC-IV Readmission		
	AUPRC (↑)	AUROC (↑)	min(+P, Se) (↑)	AUPRC (↑)	AUROC (↑)	min(+P, Se) (↑)
RETAIN	48.98 ± 1.94	77.50 ± 1.14	25.02 ± 1.40	46.71 ± 1.77	77.53 ± 0.97	35.15 ± 1.76
RNN	45.77 ± 2.13	74.34 ± 0.87	28.68 ± 1.89	48.72 ± 1.35	76.05 ± 0.81	27.12 ± 1.53
SAFARI	46.65 ± 2.47	77.11 ± 1.30	25.25 ± 1.84	45.49 ± 1.82	76.70 ± 0.95	30.69 ± 1.64
AdaCare	47.19 ± 2.40	76.97 ± 1.10	24.36 ± 1.70	46.87 ± 1.28	76.07 ± 0.84	26.40 ± 1.63
GRASP	48.36 ± 2.09	76.70 ± 0.93	18.29 ± 1.50	50.23 ± 1.50	**78.47 ± 0.88**	29.19 ± 1.37
${\mathrm{GRU}}_{\alpha }$	50.24 ± 2.08	78.36 ± 1.16	25.12 ± 1.43	50.97 ± 1.31	78.46 ± 0.86	33.80 ± 1.77
Mamba	45.98 ± 2.20	76.38 ± 1.06	24.50 ± 1.52	48.04 ± 1.42	76.87 ± 0.86	27.45 ± 1.63
${\mathrm{PathCare}}_{\mathrm{Context}-}$	47.13 ± 2.05	76.76 ± 0.92	47.17 ± 1.72	46.54 ± 1.61	76.98 ± 0.85	47.39 ± 1.41
${\mathrm{PathCare}}_{\mathrm{Gate}-}$	47.06 ± 2.11	75.43 ± 0.99	46.42 ± 1.77	50.42 ± 1.61	76.85 ± 0.88	48.61 ± 1.50
PathCare	**51.01 ± 1.90**	**78.64 ± 0.88**	**50.44 ± 1.72**	**51.52 ± 1.61**	78.41 ± 0.92	**50.30 ± 1.40**

**Note:** Bold values indicate the best performance in each column.

## Data Availability

The original contributions presented in the study are included in the article, further inquiries can be directed to the corresponding author.

## Appendix A. Online Resources

- We have released the source code at https://github.com/fuxius/PathCare (accessed on 3 April 2025).
- The MIMIC-III dataset is provided at https://physionet.org/content/mimiciii/1.4/ (accessed on 4 September 2016).
- The MIMIC-IV dataset is provided at https://physionet.org/content/mimiciv/2.2/ (accessed on 6 January 2023).
- The HM Hospitals COVID-19 Collaborator is provided at https://www.hmhospitales.com/prensa/notas-de-prensa/comunicado-covid-data-save-lives (accessed on 4 June 2024).
